# Postbiotics in Dermatology: A Literature Review of Emerging Topical Therapies for Acne, Rosacea, and Eczema

**DOI:** 10.7759/cureus.105440

**Published:** 2026-03-18

**Authors:** Julio César Flores Rodríguez, María José Morán Ortega, Alison Cristina Jimenez Ordoñez, Aylin Kerime Rojas López, Mariana Castañeda Leyva, Haizel Valencia Romero

**Affiliations:** 1 Aesthetic and Regenerative Medicine, Sociedad Mexicana de Investigación en Medicina Estética (SOMIME), Monterrey, MEX; 2 Aesthetic and Regenerative Medicine, Clínica Aura, Monterrey, MEX; 3 Aesthetic and Regenerative Medicine, Consultorio Particular Dra. María José Morán, Valle del Cauca, COL; 4 Emergency, Hospital San Rafael de Alajuela, San José, CRI; 5 Internal Medicine, Hospital General de Culiacán “Dr. Bernardo J. Gastélum”, Culiacán, MEX; 6 Aesthetic and Regenerative Medicine, Universidad Nacional Autónoma de México, Mexico City, MEX; 7 Regenerative Medicine, Universidad Nacional Autónoma de México, Mexico City, MEX

**Keywords:** acne vulgaris, atopic dermatitis, eczema, postbiotics, rosacea, skin microbiome, topical therapy

## Abstract

Postbiotics, defined as non-viable microbial cells and their metabolites, have emerged as topical therapies for inflammatory dermatoses and provide more clinical benefits than live postbiotics by eliminating infection risk and stability. This literature review aims to generalise existing knowledge about the effectiveness and mechanisms of action of first-line topical postbiotic treatments for acne, rosacea, and eczema. Search terms included "postbiotics" and keywords that represented the dermatological conditions of interest. Thematic synthesis was performed on the systematically extracted data. After screening, 16 studies were included in the review. Postbiotic preparations showed significant reductions in Scoring Atopic Dermatitis (SCORAD) and pruritus and improved barrier function in the skin, as well as longer remissions (p < 0.001). In acne vulgaris, postbiotics decreased inflammatory lesions by 50% to 70%, suppressed sebum secretion (42% to 72%), and stopped the growth of *Cutibacterium acnes*. The study concluded that topical postbiotics, reported to be effective in atopic dermatitis and acne vulgaris, have favourable safety profiles and can be integrated into treatment regimens for the aforementioned diseases. However, no interventional studies have examined rosacea, and the evidence is limited to narrative reviews in which microbiome disequilibrium remains undetermined despite a lack of clinical efficacy. Therefore, a lack of clinical trials for rosacea is a high research priority, given the substantial mechanistic rationale for this absence of high-quality pragmatic evidence.

## Introduction and background

The human skin microbiome is a complex ecological community of commensal bacteria, fungi, and viruses that plays a vital role in maintaining cutaneous health and immune homeostasis [1]. A disruption in this microbial consortium has since been recognised as a decisive pathogenic factor in a continuum of inflammatory dermatoses, including acne vulgaris, rosacea, and atopic dermatitis (eczema) [2]. Traditional treatment approaches have aimed directly at eliminating pathogenic microbes, but this therapy can be more damaging to the normal microbiota, potentially leading to prolonged gut barrier dysfunction and the emergence of antimicrobial resistance [3].

The International Scientific Association of Probiotics and Prebiotics (ISAPP) defines postbiotics as the preparations of nonliving microorganisms and/or their components that have a health benefit for the host. In contrast to live probiotics, postbiotics, such as metabolites, cell-wall fragments, enzymes, and peptides generated during the fermentation of their prebiotic counterparts by probiotics, represent a viable, stable, and non-violent option for clinical usage [4]. They have a complex mechanism of action, including antimicrobial effects against pathobionts, direct anti-inflammatory effects on immune cells and keratinocytes, strengthening the skin barrier, and shaping the resident microbiome, which makes them ideal topical treatment options [5].

The application of postbiotics in dermatology is of particular concern, given the urgent need to develop targeted, safe, and effective therapies for chronic inflammatory diseases [6]. Acne vulgaris, which is caused by the overgrowth of *Cutibacterium acnes *along with the occurrence of inflammation [7], and eczema, most commonly referring to atopic dermatitis, which is caused by disruption of the barrier and type 2 inflammation [8]. Rosacea is a chronic inflammatory skin disorder primarily affecting the central face, characterised by persistent erythema, flushing, telangiectasia, papules, and pustules. It is associated with vascular dysregulation, immune system dysfunction, and microbial factors and may also involve ocular manifestations (ocular rosacea). Metabolites are produced by probiotic bacteria, such as *Lactobacillus* [9]. *Bifidobacterium* species are a potential alternative measure to reduce cutaneous inflammation and prevent the development of pathogenic microbes while preserving homeostasis in the epidermis [10].

This review aims to generalise existing knowledge about the effectiveness and mechanisms of action of first-line topical postbiotic treatments for acne, rosacea, and eczema. The review will examine the interactions of multiple postbiotic ingredients, ranging from fermentation supernatants and isolated peptides, with the cutaneous milieu. It aims to clarify the translational relevance of postbiotics as a foundation for upcoming dermatological therapeutics and to assess their capacity to transform the impact of common dermatoses through microbial metabolites.

## Review

Methodology

The narrative review was conducted with a search of the literature on three main electronic databases: PubMed, Google Scholar, and the Cochrane Library. Search strategies used controlled vocabularies along with database-specific keywords (with Medical Subject Headings (MeSH) terms included); full search expressions are presented in Table 1. Although it is a narrative literature review, the literature search was conducted systematically using the Preferred Reporting Items for Systematic Reviews and Meta-Analyses (PRISMA) flow chart to ensure transparency and reproducibility. The reviewers included peer-reviewed English articles published between 1 January 2006 and 31 January 2026. Only randomized controlled trials, cohort trials, and review articles that tested postbiotic preparation topically in acne vulgaris, rosacea, or atopic dermatitis were considered eligible, and postbiotic constituents specifically, non-viable bacterial cells or cell lysates or metabolites, and present clinical efficacy (isolated decrease in the count of lesions, amelioration of erythema, changes in Eczema Area and Severity Index (EASI) score) or mechanistic pathways (expression of anti-inflammatory cytokines, antimicrobial activity, improvement of barrier functionality). The process of data extraction was carried out using a pre-specified narrative approach template, which includes data extraction of variables such as author(s), study design, sample characteristics, the specific postbiotic intervention, the comparator, results, and follow-up outcomes. The data collected were later analysed thematically in order to identify universal patterns of action and clinical efficacy across the three dermatology conditions, identify the current literature evidence landscape, and patterns of evidence gaps. Since the review only used published data, no ethical approval or informed consent was required.

**Table 1:** Search strings used for each information source

Database	String
PubMed	("postbiotics" OR "postbiotic compounds" OR "microbial metabolites" OR "bacterial lysates" OR "cell-free supernatant") AND ("acne" [MeSH Terms] OR "acne vulgaris" OR "rosacea" [MeSH Terms] OR "eczema" [MeSH Terms] OR "atopic dermatitis") AND ("topical" OR "skin" OR "dermatology" [MeSH Terms])
Google Scholar	("postbiotics" OR "bacterial lysate”) AND "topical therapy" AND ("acne vulgaris" OR "rosacea" OR "atopic dermatitis")
Cochrane Library	(Postbiotics OR "bacterial lysate" OR "microbial metabolite" OR "cell-free supernatant") AND (topical OR skin OR dermatology) AND (acne OR rosacea OR "atopic dermatitis" OR eczema)

Selection process of studies

**Figure 1:**
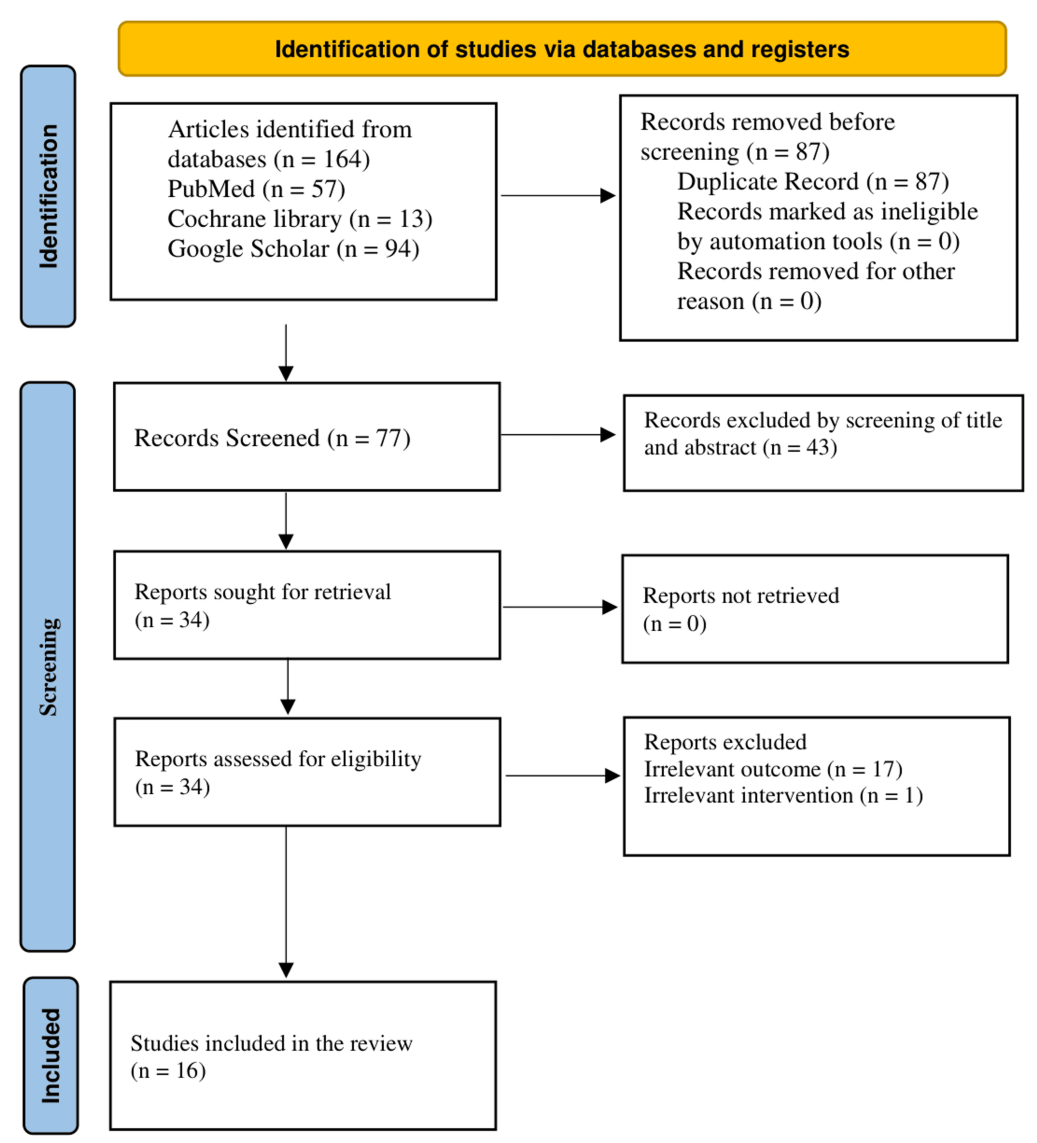
Study selection process

The initial database query provided 164 records, including 57 PubMed records, 13 Cochrane Library records, and 94 Google Scholar records. Eighty-seven duplicate records were removed prior to the screening phase. The remaining 77 unique records were screened for title and abstract, resulting in the removal of 43 articles that were evidently unrelated to the research question, such as those on gut health, oral supplementation, or non-dermatological use. The remaining 34 reports were also searched and located with success. Then these 34 reports were evaluated in detail against the eligibility criteria. After this comprehensive review of the full text, 17 reports were also eliminated based on irrelevant outcomes, and one had an irrelevant intervention. Finally, a total of 16 high-quality studies were incorporated in the final qualitative synthesis after meeting all the inclusion criteria (Figure 1).

Characteristics

Table 2 shows the key features of the 16 studies included in the literature review, and they include research conducted in 12 countries and on three continents. The studies are an uneven mix of methodology designs, with seven randomised controlled trials (RCTs), three longitudinal, two retrospective or pilot before-after studies, and two article reviews. The sizes of samples ranged between 10 and 396 responses, and the population of studies in the dermatological lifespan was indicated (between six months of age in pediatric patients and 70 years of age in adults), hence highlighting the wide applicability of postbiotic interventions to both groups. The geographic location of the research location included multicenter European trials, hospital-related studies in South Korea and China, and clinical trials in India, Indonesia, and Brazil, which represented the interest of the international scientific community in microbiome-based therapy for inflammatory skin diseases. Concerning the dermatological conditions under investigation, eight articles examined acne vulgaris, seven articles examined atopic dermatitis, and two studies provided an extensive overview of the role of the microbiome in rosacea, whereas primary interventional data on rosacea were limited to mechanisms of action as well as narrative reviews. Interventions available in the acne studies included different formulations of postbiotics, includingLactoSporin produced from the homogenate of *Enterococcus faecalis*; fermented lysates derived from various strains of *Lactiplantibacillus plantarum*; tyndallized* Lactobacillus plantarum*; and bacteriocin preparations obtained from strains of *E. faecalis*. Trials investigating atopic dermatitis were predominantly conducted using formulations containing biomass or lysates of *Vitreoscilla filiformis *and multi-species-derived postbiotic solutions from *Streptococcus. *These formulations were applied for treatment durations ranging from four to 15 weeks. Comparators varied across studies and included creams, paraffin-based moisturisers, urea-based emollients, and active pharmaceutical agents such as benzoyl peroxide; however, most single-arm studies relied on baseline comparisons to evaluate therapeutic efficacy.

**Table 2:** Characteristics of the studies included in the review SCORAD: Scoring Atopic Dermatitis; IGA: Investigator’s Global Assessment; AD: atopic dermatitis; CFU: colony-forming units; INCI: International Nomenclature of Cosmetic Ingredients; w/w: weight/weight; MTCC: Microbial Type Culture Collection; V22: VHProbi V22 strain designation; E15: VHProbi E15 strain designation; CBT SL-5: *Enterococcus faecalis* strain designation; MBPs: microbiome-based products; N/A: not applicable; yrs: years; M: male; F: female; n: number of participants

Author	Country/Setting	Study Design	Sample Size (n)	Age/Sex	Condition and Severity	Postbiotic Intervention (Strain, Formulation, Dose)	Comparator	Duration of Treatment
Gelmetti et al., 2023 [11]	8 European countries (Albania, Belarus, Croatia, Greece, Italy, Slovenia, Spain, Ukraine); multicenter	Prospective, single-arm, open-label study	396 enrolled, 324 completed	Mean age: 21.0±16.2 yrs. Age groups: 1-3 yrs (12.1%), 4-15 yrs (35.7%), ≥16 yrs (52.2%). Sex details not provided.	Mild to moderate atopic dermatitis (SCORAD)	Rilastil Xerolact PB cream (containing prebiotic alpha-glucan oligosaccharide and postbiotic *Lactobacillus* ferment). Applied twice daily.	None (single-arm study)	15 weeks (approx. three months)
Kim et al., 2024 [12]	South Korea/Three hospitals	Randomized, double-blind, vehicle-controlled, proof-of-concept trial	100 enrolled, 98 randomized (Intervention: n=65, Placebo: n=33)	12-70 years/Sex distribution not specified in extracted text, but used as an adjusted covariate	Mild-to-moderate atopic dermatitis (AD)	Topical 1.0% Strain CX postbiotic emollient. Strain CX is a solution derived from *S. pneumoniae* BF00257, *S. infantis* BF00247, and *S. mitis* BF00251.	Vehicle emollient (placebo)	Eight weeks
De et al., 2025 [13]	India (Multicenter)	Prospective, blinded, randomized comparative study	160 total (80 per group)	Age: Mean 27.16±18.49 yrs (ceramide group), 25.81±17.47 yrs (paraffin group). Sex: 85M/75F total.	Mild to moderate atopic dermatitis (IGA score < 3)	Ceramide-based postbiotic moisturiser. Applied twice daily. Strain/formulation details not specified.	Paraffin-based moisturiser. Applied twice daily.	Treatment Phase: Four weeks (both groups also used desonide cream 0.05%). Maintenance Phase: Up to three months (moisturizers only).
Seité et al. 2017 [14]	Slovakia (Private Clinic of Dermatovenerology, Svidnik); Single-center	Double-blind, randomized, comparative study	60 enrolled, 53 included at day 1	27M/33F total. Age range: 0.5 to 63 years (mean 13±14 yrs).	Moderate atopic dermatitis (SCORAD)	Emollient A (Lipikar Balm AP+) contains *Vitreoscilla filiformis* biomass grown in thermal spring water (LRP-VFB) and mannose. Applied twice daily.	Emollient B (a commercial product for AD containing triglyceride, glycerin, shea butter, and ceramide). Applied twice daily.	Four weeks (Days 1-28). Patients were pretreated for 15 days (Days -15 to 1) with drug therapy to achieve a ≥25% SCORAD reduction.
Guéniche et al., 2006 [15]	France/Monocenter (assumed, likely a research/dermatology center)	Randomized, double-blind, vehicle-controlled, intra-individual (left vs. right) trial	13	Mean age 35.7 ± 16.5 years/5 males, 8 females	Mild-to-moderate atopic dermatitis (AD) according to Rajka and Langeland criteria	Topical ointment with 5% *V. filiformis* bacterial extract (heat-treated biomass)	Vehicle ointment (base cream only)	Four weeks (twice daily application)
Prakoeswa et al. 2025 [16]	Indonesia (Dr. Soetomo General Academic Hospital, Surabaya); single-centre	Double-blind, randomized, controlled trial	60 total (30 per group)	Mean age: 36.77 yrs (Group A), 38.10 yrs (Group B). Sex: 12M/48F total (Group A: 5M/25F; Group B: 7M/23F).	Mild to moderate atopic dermatitis (SCORAD)	Emollient "Plus" (Lipikar AP+M balm) containing *V. filiformis* biomass lysate (Aqua Posae Filiformis). Applied twice daily.	Urea 10% moisturizer. Applied twice daily.	12 weeks (both groups also used standard topical steroid therapy as needed for flares).
Gueniche et al., 2008 [17]	Germany/Department of Dermatology, Eberhard Karls University Tübingen	Prospective, randomized, double-blind, placebo-controlled, parallel-group trial	75 enrolled and randomized (Verum: n=38, Placebo: n=37)	6-70 years (mean 31, median 26)/56 female, 19 male	Mild atopic dermatitis (AD) with eczematous lesions on ≤5% of total body surface. Diagnosed per Hanifin and Rajka criteria.	Topical cream containing 5% lysate of nonpathogenic gram-negative bacterium *V. filiformis*	Vehicle cream (placebo)	30 days (twice daily application)
Majeed et al., 2020 [18]	India/Monocentric (MS Clinical Research Pvt. Ltd., Bangalore)	Randomized, open-label, comparative, parallel-group trial	68 enrolled, 64 completed (LactoSporin: n=32, Benzoyl Peroxide: n=32)	18-35 years (mean ~24 years)/Both sexes (equal distribution in study population)	Mild-to-moderate acne vulgaris	Topical cream with 2% w/w LactoSporin. LactoSporin is an extracellular metabolite (postbiotic) from *Bacillus coagulans* MTCC 5856 (INCI: Bacillus ferment filtrate extract).	Topical benzoyl peroxide 2.5% gel	Three weeks (21 days)
Cui et al., 2022 [19]	China (Qingdao); single-centre	Observational, before-after study (no control group)	20 total	Age: 18-25 yrs. Sex: 10M/10F.	Mild to moderate acne vulgaris (Global Acne Assessment Scale score 2 or 3)	Anti-acne skincare cream containing 8% V22 ferment lysate (fermentation lysate of *Lactiplantibacillus plantarum* VHProbi V22). Applied twice daily.	None (baseline comparison)	Four weeks
Li et al., 2025 [20]	China/Multicenter (Guangzhou MLT Medical Cosmetic Clinic and other centers)	Retrospective self-control study	20	22-30 years (mean 25.28 ± 2.46)/3 males, 17 females	Mild-to-moderate facial acne vulgaris (Grade II-III per Chinese guidelines)	Postbiotic formulation (EVE-CHARM anti-acne lotion). Key ingredient: *Lactobacillus* fermentation lysate in a lyophilized powder mixed with a solution (contains peptides, panthenol, and hyaluronate).	None (self-control, before and after treatment)	Single treatment session, assessed four weeks post-treatment.
Cui et al., 2022 [21]	China (Jiangnan University); single-centre	Pilot, single-arm, before-after study (no control group)	22 total	Age: >16 yrs. Sex: Not specified in the main text, but Supplementary Table 1 (not provided) likely contains details.	Mild to moderate acne vulgaris (Global Acne Assessment Scale score 2 or 3)	Anti-acne lotion containing 8% ferment lysate from *L. plantarum* VHProbi E1 5. Applied twice daily.	None (baseline comparison)	Four weeks
Anwar et al. 2025 [22]	Indonesia (Rumah Sakit Universitas Hasanuddin, Makassar); single-centre	Double-blind, randomized, placebo-controlled trial	70 total (35 per group)	Age/Sex: Not specified	Acne vulgaris (severity not explicitly stated, but implied to be mild-to-moderate based on comparable studies)	Topical microbiome containing 5% *Lactococcus* ferment lysate. Formulation/dose details not specified.	Placebo	Eight weeks (implied from Figure 2 description)
Tagliolatto et al. 2020 [23]	Brazil (Private Clinic, Campinas); Single-center	Clinical, experimental, prospective, non-randomized, open-label study	28 enrolled, 10 completed	Age: 13 to 46 years (mean 29.5). Sex: 7M/21F enrolled.	Mild or moderate acne (Grade I, II, or III)	Compounded cream containing tyndallized *L. plantarum* GMNL06 (1 billion CFU/g). Applied twice daily.	None (baseline comparison)	Three months (90 days)
Han et al., 2022 [24]	South Korea/Chung-Ang University Hospital	Randomized, double-blind, placebo-controlled, split-face study	20 (all completed)	19-38 years (mean 26.5 ± 5.9)/11 males, 9 females	Mild-to-moderate acne vulgaris (IGA grade 2-3)	Topical lotion containing *Enterococcus faecalis* CBT SL-5 extract (the active ingredient is enterocin, a bacteriocin).	Vehicle lotion (identical composition but without the *E. faecalis* CBT SL-5 extract)	Four weeks (twice daily application), with a final follow-up two weeks post-treatment (Week 6).
Prajapati et al., 2025 [25]	University of South Florida	Comprehensive review	Not reported	Not reported	Rosacea	None	Not reported	Microbiome findings in rosacea • Individuals with rosacea often show dysbiosis, particularly an overgrowth of the skin mite *Demodex folliculorum*.
Rušanac et al., 2025 [26]	Croatia	Comprehensive review	Not reported	Not reported	Rosacea (characterized by facial erythema, pustules, papules, telangiectasia, and flushing)	Microbiome-based products (MBPs), including postbiotics:	Not reported	Not reported

The findings of the review are compared narratively below. However, the quantitative primary outcomes, safety outcomes, and follow-up time points are presented in Table 3.

**Table 3:** Findings of the studies included in the review SCORAD: Scoring Atopic Dermatitis; PRURISCORE: Pruritus Score; IGA: Investigator’s Global Assessment; IDQoL: Infants’ Dermatitis Quality of Life Index; CDLQI: Children’s Dermatology Life Quality Index; DLQI: Dermatology Life Quality Index; QoL: quality of life; EASI: Eczema Area and Severity Index; EASI-50: ≥50% improvement in Eczema Area and Severity Index; EASI-25: ≥25% improvement in Eczema Area and Severity Index; mEASI: modified Eczema Area and Severity Index; PVAS: Pruritus Visual Analog Scale; VAS: Visual Analog Scale; TEWL: transepidermal water loss; SCH: stratum corneum hydration; GAGS: Global Acne Grading System; SSRI: Symptom Score Reduction Index; VISIA: Canfield VISIA Skin Analysis System; AOI: area of interest; PD: phylogenetic diversity; CI: confidence interval; PP: per-protocol; PERMANOVA: permutational multivariate analysis of variance; IL-2R: interleukin-2 receptor; sIL-2R: soluble interleukin-2 receptor; IL-13: interleukin-13; IL-12: interleukin-12; IL-8: interleukin-8; IL-10: interleukin-10; TNF-α: tumor necrosis factor-alpha; CCL17: C-C motif chemokine ligand 17; CCL22: C-C motif chemokine ligand 22; LRP-VFB: La Roche-Posay *Vitreoscilla filiformis* bacteria extract; AF: affected skin; UAF: unaffected skin; *Cutibacterium acnes* (*C. acnes*); *Staphylococcus aureus* (*S. aureus*); *Staphylococcus epidermidis* (*S. epidermidis*); *Lactiplantibacillus plantarum* (*L. plantarum*); *Demodex folliculorum* (*D. folliculorum*); *Bacillus oleronius* (*B. oleronius*); *Roseomonas mucosa* (*R. mucosa*); Δ: change; p: p-value; n: number of participants; %: percentage; µg/cm^2^: micrograms per square centimeter; pH: potential of hydrogen; N/A: not applicable

Author	Primary Quantitative Outcomes	Secondary/Safety Outcomes	Follow-Up and Timepoints
Gelmetti et al., 2023 [11]	SCORAD: Mean reduction of -59.2% at week 15 (p<0.001). PRURISCORE: Mean reduction of -64.1% at week 15 (p<0.001). - IGA: Mean reduction of -49.2% at week 15 (p<0.001). - All scores showed significant improvement as early as week 5 (p<0.001).	Quality of Life: Significant improvement in all age-specific QoL indices (IDQoL, CDLQI, DLQI) from baseline to week 15 (p<0.001). Flare-ups: The mean number of recurrences during the study was 0.87, compared to an average of 3 in the same period of the previous year. Safety/Tolerability: Rated as 'very good' or 'excellent' by ~68% of patients. Adverse effects were mild (redness, tingling) and <3% discontinued. - Cosmetic Pleasantness: Rated as 'very good' or 'excellent' by >2/3 of patients.	Timepoints: t0 (baseline), t1 (5 weeks), t2 (10 weeks), and t3 (15 weeks).
Kim et al., 2024 [12]	IGA Success: % of patients with IGA score of 0/1 and ≥1-point reduction at Week 8: • Intervention: 41.5% (27/65) • Placebo: 12.1% (4/33) • p=0.005	Secondary Outcomes (at Week 8 vs. placebo): • EASI-50: 1.5% vs. 0.0% (p=0.999) • EASI-25: 46.2% vs. 9.1% (p<0.001) • Mean Δ EASI score: -0.78 vs. -0.10 (adj. diff -0.88 (95% CI -1.53 to -0.22)) • Mean Δ Skin Moisture: +9.37 vs. +4.56 (adj. diff 5.58 (95% CI 2.35 to 8.82)) • Mean Δ TEWL: -3.95 vs. +0.22 (adj. diff -6.84 (95% CI -9.76 to -3.93)) • Mean Δ Pruritus VAS: -4.83 vs. -3.19 (adj. diff -1.46 (95% CI -2.67 to -0.24)) • Mean Δ Filaggrin: +1.04 vs. +0.42 (adj. diff 0.63 (95% CI 0.01 to 1.11)) • Mean Δ Loricrin: +0.77 vs. -0.50 (adj. diff 1.27 (95% CI 0.15 to 2.40)) • Mean Δ Involucrin: +0.23 vs. -0.74 (adj. diff 0.97 (95% CI 0.04 to 1.91)) • Serum Biomarkers (PP analysis): Significant improvements in sIL-2R, IL-13, CCL17, and CCL22 for intervention vs. placebo. • Safety: No significant safety concerns reported.	Weeks 0, 4, 8, and 24 (safety follow-up)
De et al., 2025 [13]	Complete Resolution at Week 4: 100% in both groups (160/160). - Relapse Rate: 55% (44/80) in the ceramide group vs. 65% (52/80) in the paraffin group (p = 0.25). Mean Remission Period: 72.52±15.01 days (Ceramide) vs. 47.44±21.49 days (Paraffin), p = 0.0001. -Median Time to Relapse: 85 days (Ceramide) vs. 71 days (Paraffin), p = 0.05.	Safety: Both moisturizers were tolerated very well. Two patients in the ceramide group developed mild dryness during the maintenance phase, which resolved without intervention. -Efficacy Scores (Week 4): Mean IGA, EASI, VAS, and DLQI scores showed significant improvement from baseline in both groups, but the difference between groups was not statistically significant (p > 0.05).	Treatment Phase: Assessments at 2 and 4 weeks. Maintenance Phase: Telephonic follow-up every 2 weeks for a maximum of 3 months, or until relapse.
Seité et al. 2017 [14]	SCORAD Variation (Day 1 to 28): Significantly different between groups (p=0.01884). Group A (LRP-VFB): -11% vs. Group B (comparator): +35%. - Relapse Rate: 9/26 (30%) of patients in Group A worsened vs. 18/27 (60%) in Group B. - Relapse Severity: SCORAD worsening was less severe in Group A (+46%) vs. Group B (+79%).	Skin Microbiota Changes: - *Xanthomonas* genus: Significantly increased in Group A vs. Group B (p=0.0257). - *Staphylococcus* genus: Tended to increase in Group B from Day 1 to 28, but not in Group A. - Microbial differences were more pronounced in relapsing patients. - Product Usage: Average weight of product used was comparable between groups (p=0.0871).	Timepoints: Day -15 (screening), Day 1 (baseline/randomization), Day 28 (end of treatment). Skin microbiota were sampled at Day 1 and Day 28 from affected (AF) and unaffected (UAF) skin.
Guéniche et al., 2006 [15]	mEASI (Modified Eczema Area and Severity Index) at Day 28 (end of treatment): • Intervention side: 42.9% decrease from baseline • Vehicle side: 24.8% decrease from baseline • p = 0.008 (Wilcoxon signed-rank test)	Secondary Outcomes (Day 28, Intervention vs. Vehicle): • EASI: Significant decrease on intervention side (p=0.019) • Pruritus Severity Index: Significant decrease on intervention side (p=0.041) • Affected Body Surface Area: No significant difference between sides • Physician's Global Assessment: "Marked" to "Excellent" improvement (75-100% clearance) in 8/13 patients on the intervention side vs. 4/13 on the vehicle side. • Safety: No adverse events related to the study product were reported.	Weeks 0, 2, and 4 (end of treatment)
Prakoeswa et al. 2025 [16]	SCORAD: Significantly greater reduction in Group A at Weeks 8 and 12 (p≤0.05). EASI: Significantly greater reduction in Group A at Week 12 (p=0.001). - PVAS (Pruritus): Significantly greater reduction in Group A at Week 12 (p=0.001). DLQI: Significantly greater improvement in Group A at Week 12 (p=0.001).	TEWL: Significantly greater reduction in Group A at Weeks 4, 8, and 12 (p≤0.05). - Skin Hydration: Significantly greater increase in Group A at Weeks 8 and 12 (p≤0.05). - Skin pH: Significantly greater improvement (lowering) in Group A at Weeks 4, 8, and 12 (p≤0.05). - Tolerability: Both products were well-tolerated. Adverse events: 3/30 (10%) in Group A had mild, transient erythema. 8/30 (26.7%) in Group B had erythema, pruritus, and xerosis requiring antihistamines.	Timepoints: Baseline (Week 0), Week 4, Week 8, and Week 12.
Gueniche et al., 2008 [17]	SCORAD (SCORing Atopic Dermatitis) at Day 29: • Verum: Decrease from 35.39 (baseline) to 21.49 (Day 29) • Placebo: Decrease from 34.99 (baseline) to 27.93 (Day 29) • Between-group difference p = 0.0044	Secondary Outcomes (Day 29, Verum vs. Placebo): • Pruritus (VAS): Significant improvement with verum (p=0.0171). • Sleep Loss (VAS): Significant decrease from baseline only in the verum group (p=0.0074). • TEWL: Significant improvement in both groups from baseline, but no significant difference between groups. • Microflora: Tendency towards greater reduction of *S. aureus* colonization in the verum group (29.6% reduction) compared to placebo (12.0% reduction), but this was not statistically significant. • Safety: Adverse events monitored; no significant safety concerns reported.	Days 0, 15, and 29 (end of treatment)
Majeed et al., 2020 [18]	Reduction in Lesions (Day 21 vs. Baseline): • Closed Comedones:↓50.02% (p<0.0001) • Open Comedones: ↓63.11% (p=0.0069) • Papules: ↓69.96% (p<0.0001) Antera 3D (Inflammation, Day 21 vs. Baseline): • Redness: Significant ↓ (p<0.0001) • Elevation (small): ↓61.11% (p<0.0001) • Elevation (medium): ↓60.93% (p<0.0001) Sebumeter (Day 21 vs. Baseline): • Sebum secretion: ↓42.4% (from 106.74 to 61.41 µg/cm², p<0.0001)	Secondary Outcomes: • Subject Self-Assessment: Significant perceived improvement in oiliness/greasiness, pimples, acne spots, and redness. LactoSporin was perceived as significantly better than benzoyl peroxide for reducing oily/greasy skin at all timepoints (p<0.05). • Safety (Primary Irritation Patch Test): LactoSporin 2% cream was classified as "nonirritant." In the main trial, no serious adverse events or local intolerance were reported. One subject in the LactoSporin group was discontinued due to increased inflammatory lesions but recovered with rescue treatment.	Days 0, 3, 7, 14, and 21 (end of treatment)
Cui et al., 2022 [19]	Acne Lesion Proportion: Significant decrease from baseline at Week 3 and Week 4 (P<0.05). At Week 4, 70% of subjects showed a decrease. -TEWL: Significant decrease from baseline at Week 2 (P<0.05) and Week 4 (P<0.001). At Week 4, 85% of subjects showed a decrease. -Sebum Production: Significant decrease from baseline at Week 2 (P<0.05) and Week 4 (P<0.01). At Week 4, 80% of subjects showed a decrease.	Skin Tone: No statistically significant change from baseline at any time point. - Stratum Corneum Hydration (SCH): No statistically significant change from baseline at Week 2 or 4. - Skin Surface pH: No statistically significant change from baseline at Week 2 or 4. Safety: The product was found to be safe and non-irritating in human skin patch tests and acute dermal toxicity tests. No adverse effects reported. - In Vitro: *L. plantarum* VHProbi V22 inhibited *C. acnes *growth (halo of 26.33±0.58 mm).	Timepoints: Baseline (Week 0), Week 1, 2, 3, and 4 for lesion proportion and skin tone (Visia-CR). Baseline, Week 2, and Week 4 for SCH, TEWL, sebum, and pH (CK-MPA system).
Li et al., 2025 [20]	GAGS Score at Week 4: • Mean score decreased by more than half compared to baseline (p<0.01). • Symptom Score Reduction Index (SSRI): All patients achieved >26% improvement. VISIA Imaging at Week 4 (vs. Baseline): • Speckle Score: Significant improvement (p<0.05) • Pore Score: Significant improvement (p<0.05) • Red Region Score: Significant improvement (p<0.01) • Porphyrin Score: Significant improvement (p<0.01)	Secondary Outcomes: • Patient Satisfaction (at Week 4): 85% of patients were "satisfied" (35%) or "very satisfied" (50%). • Safety: Transient erythema and mild needle prick sensation were reported, which resolved within one week. No significant adverse reactions were noted.	Baseline and 4 weeks post-treatment
Cui et al., 2022 [21]	Acne Lesion Proportion: Significant decrease from baseline at Weeks 2, 3, and 4 (P<0.01). At Week 4, 59.1% of subjects showed a decrease. - TEWL: Significant decrease from baseline at Week 4 (P<0.05). 68.2% of subjects showed improvement. - Sebum Production: Significant decrease from baseline at Week 2 and Week 4 (P<0.05). At Week 4, 72.7% of subjects showed a decrease.	Pore/AOI: No statistically significant change from baseline at any time point (P>0.05). - Stratum Corneum Hydration (SCH): No statistically significant change from baseline at Week 2 or 4 (P>0.05). Skin Surface pH: No statistically significant change from baseline at Week 2 or 4 (P>0.05). Safety: Product was safe, non-irritating, and non-toxic based on microbial, heavy metal, dermal irritation, and acute toxicity tests. No adverse effects reported. - In Vitro: L. plantarum VHProbi E15 inhibited *C. acnes* growth (halo of 29.00±1.00 mm).	Timepoints: Baseline (Week 0), Weeks 1, 2, 3, and 4 for lesion proportion and pore/AOI (Visia-CR). Baseline, Week 2, and Week 4 for SCH, TEWL, sebum, and pH (CK-MPA system).
Anwar et al. 2025 [22]	Lesion Count (Treatment Group): Decreased from a mean of 48.5 (baseline) to 24.5 (post-intervention). - Sebum Levels: More effective reduction in the treatment group vs. placebo on the forehead at week 6 and on the cheeks at week 8.	Cytokine Levels (Treatment vs. Placebo): - Pro-inflammatory cytokines (IL-12, IL-8, TNF-α): Decreased in both groups, but the change was not statistically significant (P > 0.05). - Anti-inflammatory cytokine (IL-10): Increased in both groups, but the change was not statistically significant (P > 0.05).	Timepoints: Baseline (Week 0) and post-intervention (exact weeks vary by outcome: Weeks 2, 4, and 6 for lesions; Weeks 6 and 8 for sebum).
Tagliolatto et al. 2020 [23]	Clinical Response (n=10 completers): - Great improvement (>50% lesion decrease): 6/10 (60%) - Partial improvement (<50% decrease): 3/10 (30%) - No change: 0/10 (0%) - Worsening: 1/10 (10%) Overall Satisfaction: 90% (9/10) of patients who completed the study achieved a good or very good therapeutic response.	Safety: No adverse events (scaling, itching, burning, erythema) reported. Adherence/Attrition: 18/28 (64.3%) patients dropped out. Reasons: gave up without reason (12), inability to adhere to protocol (4), worsening of condition (2). One patient who dropped out for non-adherence was noted to have great improvement at Week 4.	Timepoints: Baseline (Day 0) and monthly follow-ups at Day 30, 60, and 90 (end of treatment). Clinical evaluation and photographic record at each visit.
Han et al., 2022 [24]	Investigator's Assessment of Clinical Improvement (5-point scale): • Test side score significantly higher than control at Week 2 (1.65 vs. 1.05, p=0.009), Week 4 (1.80 vs. 1.15, p=0.005), and Week 6 (1.95 vs. 1.20, p<0.0005). • Microbiome (Phylogenetic Diversity—PD): PD in skin swab samples from the test side significantly decreased at all time points vs. baseline (p<0.05). No significant decrease on the control side.	Secondary Outcomes (Test vs. Control): • Treatment Success Rate (IGA 0/1): Higher on test side (20.0%) vs. control (5.0%) at Weeks 4 & 6, but not significant (p=0.116). • Subjective Satisfaction Score: Higher on the test side at all time points, significant at Week 6 (p=0.004). • TEWL & Skin Hydration: No significant differences between groups at any time point. • Safety: The test lotion was well-tolerated with a safety profile similar to the vehicle. No treatment-related adverse events or local skin reactions were reported.	Weeks 0 (baseline), 2, 4 (end of treatment), and 6 (2 weeks post-treatment follow-up).
Prajapati et al., 2025 [25]	Therapeutic Implications (from review): • Targeting the microbiome through therapies that reduce dysbiosis (e.g., postbiotics) could offer new treatment opportunities for individuals with rosacea. • No clinical trial data on postbiotics for rosacea is presented in this review.	Not Reported	Not Reported
Rušanac et al., 2025 [26]	The review summarizes key findings on rosacea pathophysiology and potential treatment avenues but does not report quantitative outcomes from clinical trials of postbiotics for rosacea, as these are largely absent from the literature.	The review reports on the dysbiosis associated with rosacea, including: - Increased load of *Demodex folliculorum* mites. - Presence of *Bacillus oleronius* (isolated from *D. folliculorum*), which can activate proinflammatory responses. - Decreased *Roseomonas mucosa*. - Potential for *S. epidermidis* to act as a pathogen in altered skin conditions. The review mentions one case study where a patient with rosacea was treated with low-dose doxycycline alongside a probiotic (Bifidobacterium* breve* BR03 and *L. salivarius* LS01), showing significant improvement in cutaneous and ocular manifestations.	Not Reported

Efficacy of postbiotics in atopic dermatitis

The literature is consistent in showing that clinical outcomes are significantly improved in patients with atopic dermatitis treated with topical postbiotic preparations. In Gelmetti et al., it was reported that the mean Scoring Atopic Dermatitis (SCORAD) decreased by 59.2% and the Pruritus Score (PRURISCORE) decreased by 64.1% after 15 weeks of treatment using a *Lactobacillus* ferment-containing cream, and the effect was much greater in week five itself [11]. In the same vein, Kim et al. demonstrated that an animal-derived postbiotic, which is based on *Streptococcal* multi-species, was successfully used in the Investigator’s Global Assessment (IGA) in 41.5% of the patients, whereas an animal-based placebo, conversely, achieved results at most 12.1% (p=0.005), as well as with respect to pruritus, skin hydration, and transepidermal water loss (TEWL) [12]. There have always been positive results of the *V. filiformis* biomass in different types of studies: Seité et al. found that using the active form of the *V. filiformis *reduced the SCORAD by 11% in the active group and increased the SCORAD by 35% on the comparator side [14], and Gueniche et al. discovered that the use of the active type of the *V. filiformis* lowered the modified Eczema Area and Severity Index (mEASI) by 42.9% in the active group. These results were further verified by Prakoeswa et al., who found that issues with SCORAD, EASI, and pruritus scores, as well as Dermatology Life Quality Index (DLQI), reduced significantly more after *V. filiformis* lysate application than when a urea-based moisturiser was used [16]. De et al. offered relevant long-term information that proved that a ceramide-based postbiotic moisturiser had a significantly longer effect, lasting a mean remission of 72.5 days when compared to 47.4 days with a paraffin-based comparator (p=0.0001), as the percentage of complete remission between these two groups had reached 100% during the initial treatment course [13].

Efficacy of postbiotics in acne vulgaris

Findings from eight clinical trials suggest that topical postbiotic preparations yield substantial, quantifiable efficacy in acne vulgaris across a wide range of outcome measures. Majeed et al. showed a reduction of 50.02%, 63.11%, and 69.96% of closed comedones, open comedones, and papules, respectively, with a 21-day *LesoSporin* reduction of 21.45%, 21.66%, and 21.87% in *Bacillus* coagulans compared with the control, respectively [18]. It was also found that the formulation reduced sebum release by 42.4% (p<0.0001) and was rated as non-irritating in patch testing. Similar findings were obtained with *Lactiplantibacillus* ferment lysates; Cui et al. reported that in Monte Carlo by week 4, there were significant changes in the proportion of acne lesions, TEWL, and sebum production, and in vitro *C. acnes* growth was stopped [19]. Similarly, Cui et al. revealed that the proportion of lesions and sebum was significantly lowered using E15 strain lysate and that 72.7% of the test subjects exhibited reduced sebum production in week four [21]. Li et al. have indicated that the formulation of a *Lactobacillus* fermentation lysate had a reducing effect on Global Acne Grading System (GAGS) scores (more than half, p=0.01) and also had a high content of VISIA parameters, such as speckle, pore, red region, and porphyrin score (85% of patients were satisfied). Han et al. presented evidence from split-face RCTs demonstrating that *E. faecalis* CBT SL 5 extract produced significantly more clinical benefits than vehicle at weeks 2, 4, and 6, with a significant reduction in phylogenetic diversity of the skin microbiome [24]. According to Tagliolatto et al. [23], 60% of patients achieved more than 50% lesion reduction using tyndallized* L. plantarum *cream; 90% of adherents rated their therapeutic response as good or very good. Anwar et al. noted a decrease in mean lesion count from 48.5 to 24.5 after the intervention with* Lactococcus *fermentlysate, but there was no statistically significant change in cytokine [22]. This was shown by Biazzo et al., who reported mechanistic microbiome data, in which good clinical responders to a *L. plantarum*-containing formulation had stable alpha diversity, higher relative abundance of beneficial *Staphylococcus* *epidermidis, *and lower relative abundance of *C. acnes* compared with non-responders [27]. In all acne research, postbiotic preparations had positive drug safety profiles and few adverse events and therefore may be used as a well-tolerated and safe alternative or adjunct to traditional acne interventions.

Current evidence and research gaps in rosacea

Unlike the substantial clinical trial evidence for atopic dermatitis and acne vulgaris, the evidence for postbiotic interventions in rosacea is limited to mechanistic reviews and narrative syntheses, with no direct interventional evidence. A wide-ranging review of the microbiome dysbiosis of the pathogenesis of rosacea by Prajapati et al. revealed that the overgrowth of mites of the genus* Demodex folliculorum *and microbial changes were common in patients with rosacea [25]. The review posits that the cutaneous microbiome may be a new treatment target for postbiotics; however, it clearly states that interventions based on this approach have not been evaluated in clinical trials for rosacea. The dysbiotic environment of rosacea was described similarly by Rušanac et al., who found increased *Demodex* loads, immunogenic *Bacillus oleronius,* reduced *Roseomonas mucosa*, and context-dependent pathogenicity of *Staphylococcus erythropathogenes *[26]. The authors cited only one case study in which a patient with rosacea responded to low-dose doxycycline with a probiotic preparation of *Bifidobacterium breve/Lactobacillus salivarius.* Nevertheless, this is a combination therapy rather than a pure postbiotic intervention, and the evidence is at the lowest clinical level.

Clinical efficacy of topical postbiotics

The therapeutic response of topical postbiotics is durable, with patients experiencing prolonged remission and lower relapse rates than with traditional emollients [13,14]. They are improved by enhanced cutaneous barrier function, as evidenced by reduced TEWL, greater stratum corneum hydration, and a desirable modulation of the skin microbiome, specifically a reduction in pathogenic colonisation by *Staphylococcus aureus *[12,14,17]. The consistency of these results across studies, including those examining the application of *Viteascilla filiformis* and *Lactobacillus *strains conducted in different geographic regions, strengthens the evidence for the use of postbiotics in the treatment of atopic dermatitis.

Postbiotic interventions are equivalent to traditional drugs such as benzoyl peroxide for the treatment of acne vulgaris but offer the added benefit of improved tolerability and minimal irritancy [18]. In eight controlled studies, postbiotic preparations resulted in a statistically significant decrease in inflammatory and non-inflammatory lesions and a significant improvement in closed comedones (50-63%), open comedones (63%), and papules (70%). The anti-sebum properties are highly important, as the sebum synthesis decreases 42-72% [18,20,21], which is one of the major pathogenic factors in acne. The clinical observations have been validated with quantitative imaging modalities, such as VISIA and Antera3D, showing a significant reduction in erythema, lesion elevation and porphyrin fluorescence [18,20]. Antibacterial activity against *C. acnes* was also observed.

Compared with that, the postbiotic efficacy in rosacea remains purely preclinical and hypothetical. Although a putative pathophysiological model links microbial dysbiosis, such as overgrowth by the dinoflagellate and *Demodex*, with the immunogenicity of *B. oleronius,* to manifestations of rosacea [25,26], there are no RCTs or prospective cohort studies that have tested topical postbiotics specifically in rosacea samples. The available literature comprises narrative reviews and one case report on probiotic combination therapy, which does not permit a substantively meaningful conclusion regarding clinical efficacy. This gap in evidence is particularly noteworthy, given that mechanistically plausible postbiotic candidates with anti-inflammatory and microbiome-modulating properties are available and have been successful in other inflammatory dermatoses.

Limitations

This literature review has several limitations, including some studies with high attrition rates and insufficient characterisation of postbiotic interventions, as some studies provide no indication of the bacterial strain, fermentation conditions, processing methods, or concentrations of bioactive compounds. Most studies did not follow up long enough to establish the longevity of response or the safety of long-term relapse. A key limitation of this review is the limited number of studies evaluating postbiotics in rosacea compared to acne and eczema. This imbalance restricts the strength and generalisability of conclusions regarding their efficacy in rosacea.

Recommendations

Considering the identified evidence gaps, several recommendations can inform future studies. Future studies should focus on rosacea, using outcome measures and extended follow-up to assess the strength of the reaction. Second, studies should include a complete description of the bacterial strain, fermentation conditions, inactivation strategy, concentration, and bioactive components, thereby improving reproducibility. Future studies should include the pediatric population, as atopic dermatitis is more common among them.

## Conclusions

The study concluded that topical postbiotics demonstrated substantial improvements in severity scores, a balanced profile of inflammatory mediators, increased skin-barrier efficacy, reduced pathogenic microbial colonisation, and an excellent safety and tolerability profile. These findings support the use of postbiotics as a supportive or, at a minimum, complementary component of the treatment plan in the future.

## References

[1] Arora R, Kaur R, Babbar R, Dhingra S, Dhingra AK, Grewal AS (2024). Evolving advances in the cosmetic use of probiotics and postbiotics: health, regulatory and marketing aspects. Curr Pharm Biotechnol.

[2] Baldwin H, Aguh C, Andriessen A (2020). Atopic dermatitis and the role of the skin microbiome in choosing prevention, treatment, and maintenance options. J Drugs Dermatol.

[3] Choy CT, Chan UK, Siu PL (2023). A novel E3 probiotics formula restored gut dysbiosis and remodelled gut microbial network and microbiome dysbiosis index (MDI) in southern Chinese adult psoriasis patients. Int J Mol Sci.

[4] Coppola S, Avagliano C, Sacchi A (2022). Potential clinical applications of the postbiotic butyrate in human skin diseases. Molecules.

[5] De Almeida CV, Antiga E, Lulli M (2023). Oral and topical probiotics and postbiotics in skincare and dermatological therapy: a concise review. Microorganisms.

[6] Wollenberg A, Giménez-Arnau AM, Stennevin A, Ortiz-Brugués A (2024). Real-world effectiveness and tolerability of a cream containing postbiotic Aquaphilus dolomiae extract-G3 in subjects with sensitive facial skin. Eur J Dermatol.

[7] Tanojo N, Citrashanty I, Utomo B, Listiawan Y, Ervianti E, Damayanti Damayanti, Sawitri Sawitri (2023). Oral postbiotics derived from Lactobacillus sp. in treatment of atopic dermatitis: a meta-analysis. Acta Dermatovenerol Alp Pannonica Adriat.

[8] Hülpüsch C, Rohayem R, Reiger M, Traidl-Hoffmann C (2024). Exploring the skin microbiome in atopic dermatitis pathogenesis and disease modification. J Allergy Clin Immunol.

[9] Sun C, Zhu J, Sun X, Zhang Z, Sun Y, Jin Y, Wu T (2025). Targeting the human gut microbiome: a comparative review of probiotics, prebiotics, synbiotics, and postbiotics. J Adv Res.

[10] McLoughlin IJ, Wright EM, Tagg JR, Jain R, Hale JD (2022). Skin microbiome-the next frontier for probiotic intervention. Probiotics Antimicrob Proteins.

[11] Gelmetti C, Rigoni C, Cantù AM (2023). Topical prebiotics/postbiotics and PRURISCORE validation in atopic dermatitis. International study of 396 patients. J Dermatolog Treat.

[12] Kim MS, Kim HJ, Kang SM (2024). Efficacy and safety of topical Streptococcus postbiotic emollient in adolescents and adults with mild-to-moderate atopic dermatitis: a randomized, double-blind, vehicle-controlled trial. Allergy.

[13] De A, Halder S, Madan A (2025). A comparative, multicenter, prospective, randomized study to evaluate the efficacy, safety, and delay of relapse of ceramide-based post-biotic moisturizer versus paraffin-based moisturizer in mild to moderate atopic dermatitis. Cureus.

[14] Seité S, Zelenkova H, Martin R (2017). Clinical efficacy of emollients in atopic dermatitis patients - relationship with the skin microbiota modification. Clin Cosmet Investig Dermatol.

[15] Guéniche A, Hennino A, Goujon C (2006). Improvement of atopic dermatitis skin symptoms by Vitreoscilla filiformis bacterial extract. Eur J Dermatol.

[16] Prakoeswa CR, Huda BK, Indrawati D (2025). Effectiveness and tolerability of an emollient “plus” compared to urea 10% in patients with mild-to-moderate atopic dermatitis. J Cosmet Dermatol.

[17] Gueniche A, Knaudt B, Schuck E (2008). Effects of nonpathogenic gram-negative bacterium Vitreoscilla filiformis lysate on atopic dermatitis: a prospective, randomized, double-blind, placebo-controlled clinical study. Br J Dermatol.

[18] Majeed M, Majeed S, Nagabhushanam K, Mundkur L, Rajalakshmi HR, Shah K, Beede K (2020). Novel topical application of a postbiotic, LactoSporin®, in mild to moderate acne: a randomized, comparative clinical study to evaluate its efficacy, tolerability and safety. Cosmetics.

[19] Cui H, Feng C, Guo C, Duan Z (2022). Development of novel topical anti-acne cream containing postbiotics for mild-to-moderate acne: an observational study to evaluate its efficacy. Indian J Dermatol.

[20] Li Z, Li P, Xu Y (2025). Efficacy of a postbiotic formulation combined with microneedling for mild-to-moderate acne: a self-control study. J Cosmet Dermatol.

[21] Cui H, Guo C, Wang Q, Feng C, Duan Z (2022). A pilot study on the efficacy of topical lotion containing anti-acne postbiotic in subjects with mild-to-moderate acne. Front Med (Lausanne).

[22] Anwar AI, Zainuddin AA, Hutabarat EN, Savitri KAP (2025). Effectiveness of topical microbiome containing Lactococcus ferment lysate 5% in acne vulgaris patients. J Pak Assoc Dermatol.

[23] Tagliolatto S, França PF, dos Santos KMP (2020). Use of topical tyndallized probiotic bacteria in the treatment of acne vulgaris. Surg Cosmet Dermatol.

[24] Han HS, Shin SH, Choi BY (2022). A split face study on the effect of an anti-acne product containing fermentation products of Enterococcus faecalis CBT SL-5 on skin microbiome modification and acne improvement. J Microbiol.

[25] Prajapati SK, Lekkala L, Yadav D, Jain S, Yadav H (2025). Microbiome and postbiotics in skin health. Biomedicines.

[26] Rušanac A, Škibola Z, Matijašić M, Čipčić Paljetak H, Perić M (2025). Microbiome-based products: therapeutic potential for inflammatory skin diseases. Int J Mol Sci.

[27] Biazzo M, Pinzauti D, Podrini C (2025). SkinDuoTM as a targeted probiotic therapy: shifts in skin microbiota and clinical outcomes in acne patients. Int J Mol Sci.

